# DeepRank-GNN-esm: a graph neural network for scoring protein–protein models using protein language model

**DOI:** 10.1093/bioadv/vbad191

**Published:** 2024-01-05

**Authors:** Xiaotong Xu, Alexandre M J J Bonvin

**Affiliations:** Department of Chemistry, Faculty of Science, Computational Structural Biology Group, Bijvoet Centre for Biomolecular Research, Utrecht 3584 CS, The Netherlands; Department of Chemistry, Faculty of Science, Computational Structural Biology Group, Bijvoet Centre for Biomolecular Research, Utrecht 3584 CS, The Netherlands

## Abstract

**Motivation:**

Protein–Protein interactions (PPIs) play critical roles in numerous cellular processes. By modelling the 3D structures of the correspond protein complexes valuable insights can be obtained, providing, e.g. starting points for drug and protein design. One challenge in the modelling process is however the identification of near-native models from the large pool of generated models. To this end we have previously developed DeepRank-GNN, a graph neural network that integrates structural and sequence information to enable effective pattern learning at PPI interfaces. Its main features are related to the Position Specific Scoring Matrices (PSSMs), which are computationally expensive to generate, significantly limits the algorithm's usability.

**Results:**

We introduce here DeepRank-GNN-esm that includes as additional features protein language model embeddings from the ESM-2 model. We show that the ESM-2 embeddings can actually replace the PSSM features at no cost in-, or even better performance on two PPI-related tasks: scoring docking poses and detecting crystal artifacts. This new DeepRank version bypasses thus the need of generating PSSM, greatly improving the usability of the software and opening new application opportunities for systems for which PSSM profiles cannot be obtained or are irrelevant (e.g. antibody-antigen complexes).

**Availability and implementation:**

DeepRank-GNN-esm is freely available from https://github.com/DeepRank/DeepRank-GNN-esm.

## 1 Introduction

Protein–protein interactions (PPIs) are essential for many biological processes. Obtaining 3D structural information on the corresponding assemblies is key to uncover their functions, and misfunctions in case of disease. Experimentally, this is typically done by cryo-electron microscopy or tomography, X-ray crystallography or, to a less extent, Nuclear Magnetic Resonance spectroscopy. This is, however, often a labour-intensive and costly process. As a result, computational approaches, often supplemented by a limited amount of data, have emerged as valuable alternatives. This has been done classically by docking and/or integrative modelling approaches (Koukos and Bonvin 2020, van Noort *et al.* 2021, Braberg *et al.* 2022). Artificial Intelligence (AI) has also entered the field of complex prediction, in particular with various applications of AlphaFold2 (Jumper *et al.* 2021) and Alpha Fold-multimer (Evans *et al.* 2022) to the prediction of protein–protein (Zhu *et al.* 2023, Gao *et al.* 2022) and protein-peptide (Johansson-Åkhe and Wallner 2022, Tsaban *et al.* 2022, Chang and Perez 2023) complexes.

One of the challenges in computational approaches is accurately identifying near-native PPI conformations from the large pool of generated models, which is often referred to as ‘scoring’. Multiple in-silico approaches have been proposed to this end (Xue *et al.* 2015, Geng *et al.* 2019, Casadio *et al.* 2022), including physics-based scoring function implemented, e.g. in HADDOCK (Dominguez *et al.* 2003), knowledge-based statistical potentials such as GOAP (Zhou and Skolnick 2011), classical machine learning methods such as iScore (Geng *et al.* 2020), meta predictors (Jung *et al.* 2023) and, in recent years, deep learning approaches such as DOVE (Wang *et al.* 2020), DeepRank (Renaud *et al.* 2021), GNN-DOVE (Wang *et al.* 2021) and a Graph Neural Network (GNN) version of DeepRank, DeepRank-GNN (Réau et al. 2023), which was shown to have the best performance at the time of publication. It was recently applied to the classification of physiological versus non-physiological interfaces in homomeric complexes, showing the best performance of all single predictions (Schweke *et al.* 2023).

DeepRank-GNN converts 3D structures of PPI complexes into residue-level graphs and uses the resulting graph interaction networks for making predictions. It was trained on two tasks: classification of crystallographic interfaces and scoring of PPI complexes. In both cases, the Position-specific Scoring Matrices (PSSMs) were found to be the main features driving the predictions. PSSMs provide valuable information about the evolutionary conservation profiles of residues at interfaces of PPIs, helping to identify functionally important residues. However, computing PSSMs is computationally expensive, particularly when aiming at larger alignment depths that enhance the results’ reliability. This requirement, coupled with the need for a non-redundant protein sequence database, can pose challenges for users of the software and impact its overall usability. Despite the availability of pre-computed PSSMs at external databases such as 3DCONS (Sanchez-Garcia *et al.* 2017) and Conserved Domain Database (Lu *et al.* 2020), they often have limitations in their content. Therefore, there is a need to find more flexible and fast way to compute features to enhance the usability of DeepRank-GNN.

The scaling of large language models (LLMs) to incorporate billions or even trillions of training parameters has unlocked unprecedented capabilities, enabling advanced reasoning and the generation of lifelike images and text (Wei *et al.* 2022). This transformative progress in natural language processing has not only revolutionized the field but has also paved the way for the development of protein language models. Evolutionary Scale Modeling-2 (ESM-2) is a state-of-the-art transformer architecture trained on millions unique protein sequences to predict the identity of randomly masked amino acids (Lin *et al.* 2023). By leveraging a massive-scale training approach that involves solving missing puzzles with over 15 billion parameters, ESM-2 is able to effectively internalize complex sequence patterns across evolution and generate high-quality embeddings that are rich in both evolutionary and functional insights. ESM-2 embeddings, which are very fast to compute, are therefore valuable for various protein-related tasks, such as structure prediction, design, and functional annotation (Lin *et al.* 2023).

To bypass the lengthy computation of PSSMs, we present here DeepRank-GNN-esm, a new version of our DeepRank-GNN algorithm that incorporates ESM-2 embeddings. The process of generating ESM-2 embeddings for a protein sequence is significantly more efficient in terms of computational resources and time investment as it does not rely on multiple sequence alignments (MSAs). We show that those embeddings can substitute the PSSM features at no loss in performance. By integrating ESM-2 embeddings into DeepRank-GNN, we achieve a significant acceleration in PPI scoring tasks, resulting in improved usability without compromising the performance. We also show that combining PSSMs and ESM-2 embedding does lead to an improvement in overall scoring performance, indicating that they do contain complementary information. Our algorithm is freely available as a Python package (https://github.com/DeepRank/DeepRank-GNN-esm), featuring two re-trained models, making it easily accessible for researchers in the field of structural biology and bioinformatics.

## 2 Methods

### 2.1 Dataset

#### 2.1.1 BM5 and CAPRI datasets

We used the same training and test dataset as previously used for DeepRank and DeepRank-GNN and available from the SBGRID data repository (Meyer *et al.* 2016) (https://doi.org/10.15785/SBGRID/843). It consists of docked models of 143 complexes from the Docking benchmark dataset version 5 (BM5) (Vreven *et al.* 2015), excluding antibody-antigen complexes and complexes with more than two chains. To address potential variations in model performance arising from the selection of data subsets for training, as noted by (Réau *et al.* 2023), we randomly divided the 128 complexes and their associated models into training (80%) and evaluation (20%) datasets by complex for cross-validation while reserving 15 complexes as the independent test set. The final models were obtained by training on the full training dataset as aligned with the training methodology used in the final model of DeepRank-GNN. Additionally, as an independent test set, we took 13 complexes from the CAPRI score set (Lensink and Wodak 2014) as described in DeepRank and DeepRank-GNN, details of which can be found in Supplementary Table S1, to further validate our algorithm's performance.

#### 2.1.2 MANY/DC benchmark

We explored the application of our proposed models on the task of detecting crystal artifacts from true biological interfaces. Classification of biological or crystallographic PPIs is challenging. In a recent community-wide investigation on assigning protein complexes to correct oligomeric state, DeepRank-GNN achieved the highest AUC among 252 scoring functions (Schweke *et al.* 2023). To further investigate the efficacy of ESM features, we trained two binary classification models on the MANY dataset (Baskaran *et al.* 2014) and assessed their performances on the DC dataset (Duarte *et al.* 2012), following the methodology employed by DeepRank and DeepRank-GNN. The MANY/DC benchmark is accessible from https://doi.org/10.15785/SBGRID/843.

### 2.2 Graph generation

To construct the protein graphs, we selected protein residues located within 8.5 Å of the interface of the complex as graph nodes. Interface edges and internal edges of the graphs were defined as previously described (Réau *et al.* 2023). Node and edge features were computed and stored in HDF5 format for efficient processing. We calculated ESM-2 embeddings for each protein sequence with model and scripts provided by Lin et al. (2023) and assigned the embedding for each residue to the corresponding graph node. Details of the ESM-2 embeddings computation can be found in the Supplementary Method S1 section.

### 2.3 Model training

DeepRank-GNN (Réau *et al.* 2023) is structured around a series of Graph Convolution Layers (GCLs), activation layers, and pooling layers. Two GCLs with attention mechanism are applied separately to the interface graphs and the internal graphs. Pooling layers serve the dual purpose of reducing node count and extracting higher-level features. This is achieved through the implementation of clustering algorithms, followed by a max pooling step.

We adapted the model architecture of DeepRank-GNN (Réau *et al.* 2023) and trained three algorithms (DeepRank-GNN-esm-pssm, DeepRank-GNN-esm and DeepRank-GNN-no-pssm) to predict the fraction of native contacts (*f*_nat_) for PPI conformations. The various features used in each model are listed in Table 1. During model training, we used a mean squared error loss function, optimized the models using the Adam algorithm with a batch size of 128 and a learning rate of 0.001. We trained all models for 20 epochs. Supplementary Figures S1–S3 show the training and validation loss curves, along with the corresponding AUC values, for both the final models trained on the complete dataset and the models generated during cross-validation.

**Table 1.** Model features and the number of total trainable parameters.

Features^a^	Dimension	Model^b^			
		1	2	3	4
Type	20	√	√	√	√
Polarity	4	√	√	√	√
BSA	1	√	√	√	√
Charge	1	√	√	√	√
Cons	1	√		√	
PSSM_IC	1	√		√	
PSSM	20	√		√	
Embedding	1280	√	√		
Distance	1	√	√	√	√
Total trainable parameters		52169	51465	10505	11209

a Type represents the amino acid type, BSA the buried surface area of the complex, Cons the Conservation score (from PSSM), PSSM IC the Information content (from PSSM), PSSM the Position-specific scoring matrix, distance the normalized distance between graph nodes.

b Model 1: DeepRank-GNN-esm-pssm, Model 2: DeepRank-GNN-esm, Model 3: DeepRank-GNN, and Model 4: DeepRank-GNN-no-pssm.

## 3 Results

### 3.1 Combining PSSMs and ESM embeddings improves the scoring performance

#### 3.1.1 Performance of 10-fold cross-validation on the BM5 evaluation set

We trained DeepRank-GNN-esm-pssm, which combines both PSSM and ESM embeddings as features, using 10-fold cross-validation on the BM5 training set. The average AUC obtained across all folds on their evaluation set is 0.639 ± 0.054 (see Supplementary Fig. S1).

The model performance of the final DeepRank-GNN-esm-pssm model trained on the full training set is evaluated on the independent validation set using AUC and six machine learning metrics: Precision, MCC (Matthews's correlation coefficient), F1, Recall, *R*^2^, and Pearson correlation coefficient (see Supplementary Method S2 for their definitions). DeepRank-GNN-esm-pssm model has the highest AUC (0.95) during training (Supplementary Fig. S4). Since the dataset is highly imbalanced, we focus our discussion on the F1 score and MCC. The training results show that the DeepRank-GNN-esm-pssm model, which combines PSSMs and ESM embeddings, outperformed the original DeepRank-GNN model with a 7.59% increase in F1 and an 8.23% increase in MCC (Table 2). Moreover, the DeepRank-GNN-esm-pssm model shows a stronger correlation between predicted *f*_nat_ values and the ground truth than the original model, as indicated by a higher *R*^2^ value (0.916 versus 0.826). This enhanced correlation is clearly visible in the scatter plot in Supplementary Fig. S5.

**Table 2.** Comparison of model performances on the BM5 evaluation set.

Metrics^a^	Model^b^			
	1	2	3	4
Precision	**0.83**	0.824	0.778	0.685
MCC	**0.776**	0.771	0.717	0.655
Recall	**0.758**	0.754	0.701	0.682
F1	**0.793**	0.788	0.737	0.683
*R* ^2^	0.916	**0.921**	0.826	0.683
Pearson_r	0.957	**0.96**	0.909	0.842
AUC	**0.872**	0.87	0.842	0.827
Success rate % (Top1)	**89.8**	**89.8**	85.8	80.3
Success rate % (Top5)	**97.6**	96.1	96.1	92.1
Success rate % (Top20)	**100**	97.6	98.4	96.9
Success rate % (Top50)	**100**	100	99.2	98.4

a A true positive is defined as a model with *DockQ* > 0.23. The best value for each metric is marked in bold.

b Model 1: DeepRank-GNN-esm-pssm, Model 2: DeepRank-GNN-esm, Model 3: DeepRank-GNN, and Model 4: DeepRank-GNN-no-pssm.

The enhanced performance on the validation set can be attributed to two factors. Firstly, the DeepRank-GNN-esm-pssm model uses the ESM embeddings, which provide both more node features (1328) and additional information. The larger feature size results in an increased number of learnable parameters, enhancing the model's ability to generalize patterns from the input data (Table 1).

#### 3.1.2 Performance on the BM5 and CAPRI test sets

**Figure 1.**
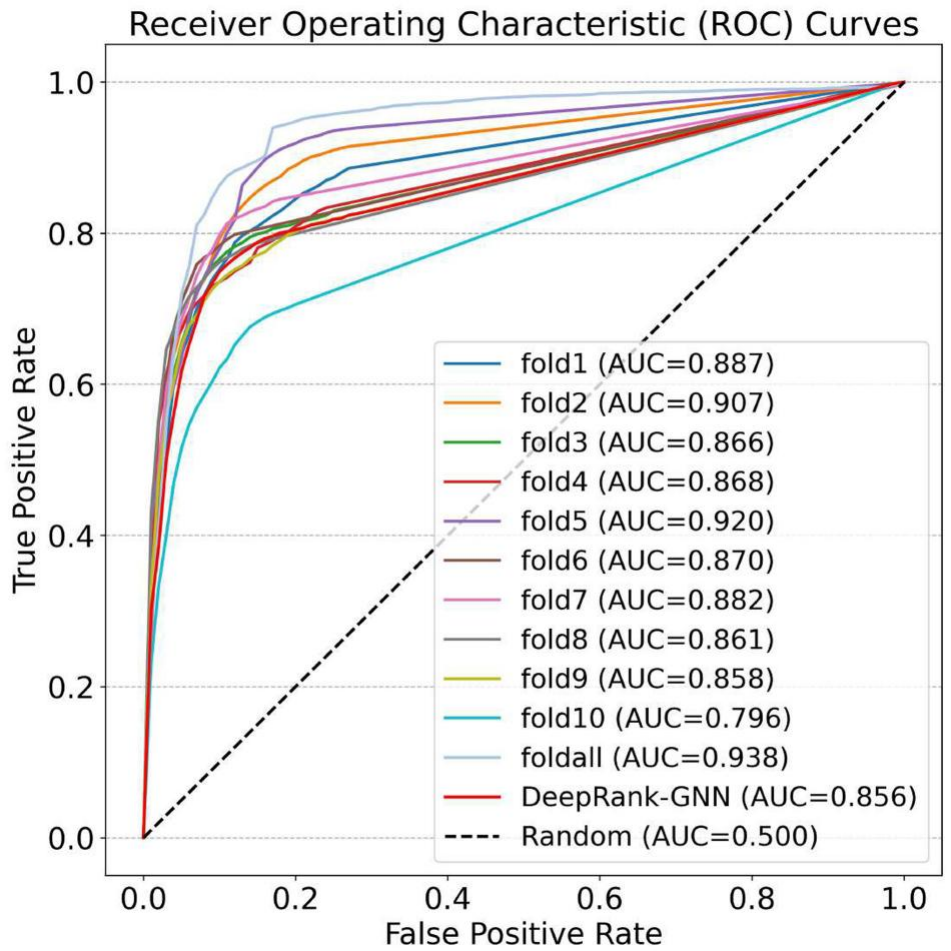
Receiver operating characteristic curves of DeepRank-GNN-esm-pssm model on the BM5 test set (a true positive is defined as a model with *DockQ* > 0.23).

We first evaluated all 11 DeepRank-GNN-esm-pssm models on the independent BM5 test set by computing the AUC using a DockQ threshold of 0.23 to distinguish between good and bad models (the model outputs a continuous scale output between 0 and 1). To create a smoother curve, the true positive rate (TPR) values are estimated at particular false positive rate (FPR) values using interpolation. The results, depicted in Fig. 1, demonstrate that the final model (foldall) achieves the highest AUC value (0.938) compared to all models obtained in cross-validation. Interestingly, nine models trained on subsets of the data outperforms the original DeepRank-GNN model. The variation in performance between folds also highlights the dataset dependency of the algorithm. To be aligned with DeepRank-GNN, we selected the final model trained on the full dataset for subsequent analysis.

As the second test set, to ensure a fair comparison of the model’s performance outside of BM5, we compared it with six other scoring methods on the CAPRI score set, including DeepRank (Renaud *et al.* 2021), DeepRank-GNN (Réau *et al.* 2023), GNN_DOVE (Wang *et al.* 2021), HADDOCK (Dominguez *et al.* 2003), iScore (Geng *et al.* 2020), and GOAP (Zhou and Skolnick 2011). As metric for the comparison, we use the per-complex success rate, which is calculated by counting the number of docking cases in which at least one near-native model is found among the top-k ranking models, divided by the total number of cases. Table 3 demonstrates the superior performance of DeepRank-GNN-esm-pssm over DeepRank-GNN, particularly in relation to the top five ranks. DeepRank-GNN-esm-pssm achieves a 30.8% success rate in correctly identifying the structures, surpassing DeepRank-GNN (23.1%). While DeepRank-GNN shows superior performance in scoring near-native conformations when assessing Top50 models, we consider the performance at earlier ranks to be more crucial for PPI scoring tasks.

**Table 3.** Comparison of model on the CAPRI Score set.

Algorithm^a^	AUC	Success rates (%)			
		Top1	Top5	Top20	Top50
Model1	0.78	15.4	30.8	46.2	53.8
Model2	**0.79**	15.4	30.8	46.2	**69.2**
Model3	0.71	7.7	23.1	46.2	61.5
Model4	0.66	7.7	30.8	46.2	46.2
DeepRank	0.59	15.4	15.4	53.8	61.5
HADDOCK	0.55	23.1	23.1	46.2	61.5
iScore	0.64	**38.5**	46.2	53.8	**69.2**
GOAP	0.42	0	23.1	46.2	53.8
GNN-Dove	0.54	15.4	**53.8**	**69.2**	**69.2**

a Model 1: DeepRank-GNN-esm-pssm, Model 2: DeepRank-GNN-esm, Model 3: DeepRank-GNN, and Model 4: DeepRank-GNN-no-pssm. The best value for each metric is marked in bold.

These results demonstrate the improved scoring performance achieved by combining PSSMs and ESM-2 embeddings in the DeepRank-GNN architecture.

### 3.2 ESM embeddings can substitute PSSM features without any performance loss

#### 3.2.1 Performance of 10-fold cross-validation on the BM5 evaluation set

To further explore the feasibility of substituting the PSSM features with ESM embeddings, we compared the performance of a modified version of our model, termed DeepRank-GNN-esm, which excludes all PSSM-related features (PSSM, PSSM-IC, and Cons). Despite not reaching the same performance level as the DeepRank-GNN-esm-pssm model, the DeepRank-GNN-esm model demonstrated superiority over the original DeepRank-GNN model, with a 7.56% increase in recall and a 7.53% increase in MCC (Table 2). These findings indicate that PSSM features can be substituted by ESM embeddings as an effective alternative which does not require the more costly computation of the PSSM.

Both PSSM and ESM embeddings have the inherent capacity to capture evolutionary conservation, enabling the detection of functionally important residues. ESM embeddings offer a substantial increase in information compared to PSSMs, both in terms of the database involved in the computation process and the dimensionality of the features. This is attributed to the fact that PSSM features, with 20 features per residue, are derived from sequence alignment with the non-redundant (NR) database, whereas ESM embeddings, with 1280 features per residue, are obtained from training on a significantly larger volume of sequences (∼138 million sequences). This distinction potentially explains the effectiveness of substituting PSSM feature with the embeddings. In contrast, the DeepRank-GNN-no-pssm model, which excludes both PSSM and ESM embeddings, has high training loss even after 20 epochs (Supplementary Fig. S4), which indicates its limited ability to effectively learn from the input data. This model also shows the lowest performance across all metrics.

#### 3.2.2 Performance on the BM5 and CAPRI test sets

Our comparison of model performance (Table 4) on the BM5 test set demonstrates that the DeepRank-GNN-esm model outperforms the original DeepRank-GNN model in terms of overall AUC. The DeepRank-GNN-no-pssm model, which excludes both PSSM and ESM-2 embeddings, exhibits the lowest AUC value (0.78). Within Top50 ranks, both models with protein language embeddings are able to identify correct conformations for 13 targets, surpassing the original DeepRank-GNN model by over 10%.

**Table 4.** Comparison of model performances on the BM5 test set.

Algorithm^a^	AUC	Success rates (%)			
		Top1	Top5	Top20	Top50
Model1	0.81	60	73.3	**80**	**86.7**
Model2	**0.87**	**66.7**	**80**	**80**	**86.7**
Model3	0.7	60	66.7	66.7	73.3
Model4	0.78	60	66.7	73.3	73.3

a Model 1: DeepRank-GNN-esm-pssm, Model 2: DeepRank-GNN-esm, Model 3: DeepRank-GNN, and Model 4: DeepRank-GNN-no-pssm. The best value for each metric is marked in bold.

**Figure 2.**
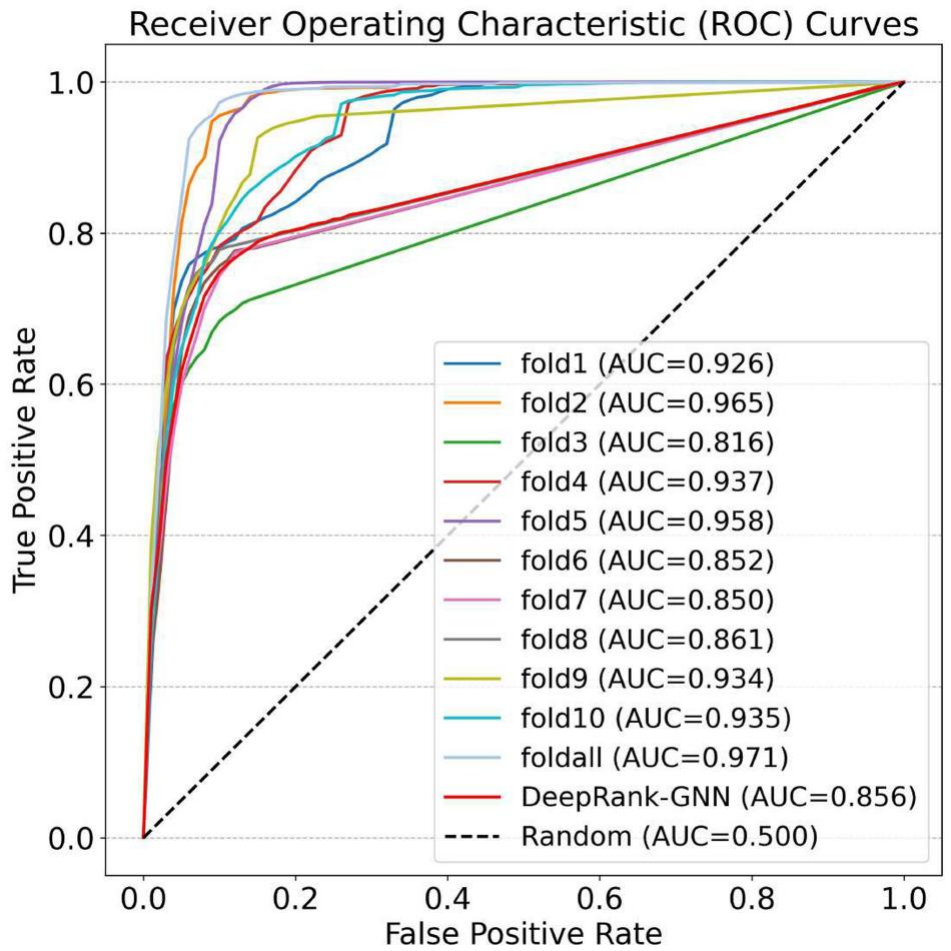
Receiver operating characteristic curves of DeepRank-GNN-esm model on the BM5 test set (a true positive is defined as a model with *DockQ* > 0.23).

The AUC curves of 11 DeepRank-GNN-esm models on BM5 test set are plotted in Fig. 2.

In the CAPRI score set, the DeepRank-GNN-esm model exhibits the highest predictive performance (69.2% top50), in par with iScore and GNN-Dove, even surpassing the DeepRank-GNN-esm-pssm model. Within the top 50 ranks, the DeepRank-GNN-esm model successfully identifies correct complex conformations for nine out of 13 targets. DeepRank-GNN, DeepRank and HADDOCK closely follow with success rates of 61.5% (Table 3). Notably, six graph-based approaches, including DeepRank-GNN and its three derivatives, iScore, and GNN_Dove, consistently demonstrate higher success rates at earlier ranks, distinguishing themselves from the other methods. iScore performs well at rank 1, while the DeepRank-GNN-esm model excels at later ranks. Combining the DeepRank-GNN-esm model with iScore predictions could potentially yield even better predictions.

There are two plausible explanations for the observed superior performance of DeepRank-GNN-esm over DeepRank-GNN-esm-pssm. Both ESM-2 embeddings and PSSM capture evolutionary information, employing them simultaneously may lead to data redundancy and amplify noise within the data. Further, the larger set of features used by DeepRank-GNN-esm-pssm increases the complexity and the number of trainable parameters. This can make convergence more challenging, as evidenced by the higher overall training loss after training for 20 epochs (Supplementary Fig. S1).

#### 3.2.3 Application of DeepRank-GNN-esm model for discriminating physiological from non-physiological interfaces

Next to scoring, we also trained our models on the task of discriminating biological interfaces from crystallographic ones using the MANY dataset (Baskaran *et al.* 2014) for training and the DC dataset (Duarte *et al.* 2012) as independent test set. During the training process, we monitored the loss curves and calculated AUC values (Supplementary Fig. S6). In contrast to the scoring dataset, in this case both the training and test datasets are perfectly balanced and the accuracy is therefore a suitable metric for comparing the performance of the models. Both DeepRank-GNN-esm-pssm and DeepRank-GNN-esm achieve an accuracy of 0.83 (Table 5), comparable to the reported accuracy of DeepRank-GNN (0.82) on the DC test dataset. Our results demonstrate the effectiveness of ESM features in accurately identifying true biological interfaces from crystal artifacts.

**Table 5.** Comparison of model performances on the DC test set.

Model^a^	Accuracy (%)
PISA	79
PRODIGY-Crystal	74
DeepRank	86
Model1	83
Model2	83
Model3	82

a Model 1: DeepRank-GNN-esm-pssm, Model 2: DeepRank-GNN-esm, Model 3: DeepRank-GNN.

### 3.3 Computational speed

The gain in efficiency of using ESM-2 embeddings compared to traditional PSSM profiles is significant: Generating a single PSSM profile requires a non-redundant protein database of 176 GB and takes approximately two hours when computed on a single core. In contrast, generating ESM-2 embeddings for the same sequence only requires a pre-trained model of 2.5 GB size and takes approximately 5 s. This represents a more than 100-fold increase in efficiency compared to the PSSM generation process. These findings are supported by data presented in Supplementary Table S2, which highlights the efficiency gains achieved by ESM-2 embeddings. Our data also indicate that there is no significant increase in model inference time associated with the use of protein language model features.

## 4 Conclusions

By integrating language model-based features into our existing deep learning framework, we have developed the DeepRank-GNN-esm algorithm, which enhances the scoring of protein–protein complexes. Adding the language model embeddings results in an increased prediction performance. Overall, our findings on two PPI-related tasks (scoring and discrimination of biological interfaces) suggest that PSSM features can be replaced by ESM-2 embeddings. This comes with the advantage of bypassing the requirement of pre-generating the PSSM, a cumbersome and computationally more expensive process, while maintaining or even slightly improving the performance. This opens new avenues for future research, particularly in the case of antibody-antigen complexes or protein-peptide complexes where PSSM profiles may not be applicable. To the best of our knowledge, DeepRank-GNN-esm is the first method to apply protein language models in graph neural networks for protein–protein models evaluation tasks.

## Supplementary Material

vbad191_Supplementary_Data

## Data Availability

Software and related data are freely available on GitHub at https://github.com/DeepRank/DeepRank-GNN-esm. BM5 docking models and CAPRI score set, MANY and DC dataset used in training and evaluation can be downloaded from https://data.sbgrid.org/dataset/843/.

## References

[vbad191-B1] Baskaran K, Duarte JM, Biyani N et al A PDB-wide, evolution-based assessment of protein–protein interfaces. BMC Struct Biol 2014;14:22. 25326082 10.1186/s12900-014-0022-0 PMC4274722

[vbad191-B2] Braberg H , Echeverria I, Kaake RM et al From systems to structure–using genetic data to model protein structures. Nat Rev Genet 2022;23:342–54. 35013567 10.1038/s41576-021-00441-w PMC8744059

[vbad191-B3] Casadio R , Martelli PL, Savojardo C. Machine learning solutions for predicting protein–protein interactions. WIREs Comput Mol Sci 2022;12:e1618.

[vbad191-B4] Chang L , Perez A. Ranking peptide binders by affinity with AlphaFold. Angew Chem 2023;135:e202213362. 10.1002/anie.202213362 36542066

[vbad191-B5] Dominguez C , Boelens R, Bonvin AMJJ et al HADDOCK: a protein−protein docking approach based on biochemical or biophysical information. J Am Chem Soc 2003;125:1731–7. 12580598 10.1021/ja026939x

[vbad191-B6] Duarte JM , Srebniak A, Schärer MA et al Protein interface classification by evolutionary analysis. BMC Bioinformatics 2012;13:334. 23259833 10.1186/1471-2105-13-334 PMC3556496

[vbad191-B7] Evans R , O’Neill M, Pritzel A et al Protein complex prediction with AlphaFold-Multimer. bioRxiv, 10.1101/2021.10.04.463034, 2022, preprint: not peer reviewed.

[vbad191-B8] Gao M , Nakajima An D, Parks JM et al AF2Complex predicts direct physical interactions in multimeric proteins with deep learning. Nat Commun 2022;13:1744. 35365655 10.1038/s41467-022-29394-2 PMC8975832

[vbad191-B9] Geng C , Xue LC, Roel-Touris J et al Finding the ΔΔG spot: are predictors of binding affinity changes upon mutations in protein–protein interactions ready for it? WIREs Comput Mol Sci 2019;9:e1410.

[vbad191-B10] Geng C , Jung Y, Renaud N et al iScore: a novel graph kernel-based function for scoring protein–protein docking models. Bioinformatics 2020;36:112–21. 31199455 10.1093/bioinformatics/btz496 PMC6956772

[vbad191-B11] Johansson-Åkhe I , Wallner B. Improving peptide-protein docking with AlphaFold-Multimer using forced sampling. Front Bioinform 2022;2:959160. 36304330 10.3389/fbinf.2022.959160 PMC9580857

[vbad191-B12] Jumper J , Evans R, Pritzel A et al Highly accurate protein structure prediction with AlphaFold. Nature 2021;596:583–9. 34265844 10.1038/s41586-021-03819-2 PMC8371605

[vbad191-B13] Jung Y , Geng C, Bonvin AMJJ et al MetaScore: a novel machine-learning-based approach to improve traditional scoring functions for scoring protein–protein docking conformations. Biomolecules 2023;13:121. 36671507 10.3390/biom13010121 PMC9855734

[vbad191-B14] Koukos PI , Bonvin AMJJ. Integrative modelling of biomolecular complexes. J Mol Biol 2020;432:2861–81. 31783069 10.1016/j.jmb.2019.11.009

[vbad191-B15] Lensink MF , Wodak SJ. Score_set: a CAPRI benchmark for scoring protein complexes. Proteins Struct Funct Bioinform 2014;82:3163–9. 10.1002/prot.24678 25179222

[vbad191-B17] Lin Z , Akin H, Rao R et al Evolutionary-scale prediction of atomic-level protein structure with a language model. Science 2023;379:1123–30. 36927031 10.1126/science.ade2574

[vbad191-B18] Lu S , Wang J, Chitsaz F et al CDD/SPARCLE: the conserved domain database in 2020. Nucleic Acids Res 2020;48:D265–8. 31777944 10.1093/nar/gkz991 PMC6943070

[vbad191-B19] Meyer PA , Socias S, Key J et al Data publication with the structural biology data grid supports live analysis. Nat Commun 2016;7:10882. 26947396 10.1038/ncomms10882 PMC4786681

[vbad191-B20] Réau M , Renaud N, Xue LC et al DeepRank-GNN: a graph neural network framework to learn patterns in protein–protein interfaces. Bioinformatics 2023;39:btac759. 36420989 10.1093/bioinformatics/btac759 PMC9805592

[vbad191-B21] Renaud N , Geng C, Georgievska S et al DeepRank: a deep learning framework for data mining 3D protein–protein interfaces. Nat Commun 2021;12:7068. 34862392 10.1038/s41467-021-27396-0 PMC8642403

[vbad191-B22] Sanchez-Garcia R , Sorzano C, Carazo J et al 3DCONS-DB: a database of position-specific scoring matrices in protein structures. Mol Basel Switz 2017;22:2230. 10.3390/molecules22122230 PMC6149929 29244774

[vbad191-B23] Schweke H , Xu Q, Tauriello G et al Discriminating physiological from non-physiological interfaces in structures of protein complexes: a community-wide study. Proteomics 2023;23:e2200323. 37365936 10.1002/pmic.202200323 PMC10937251

[vbad191-B24] Tsaban T , Varga JK, Avraham O et al Harnessing protein folding neural networks for peptide–protein docking. Nat Commun 2022;13:176. 35013344 10.1038/s41467-021-27838-9 PMC8748686

[vbad191-B25] van Noort CW , Honorato RV, Bonvin AMJJ et al Information-driven modeling of biomolecular complexes. Curr Opin Struct Biol 2021;70:70–7. 34139639 10.1016/j.sbi.2021.05.003

[vbad191-B26] Vreven T , Moal IH, Vangone A et al Updates to the integrated protein–protein interaction benchmarks: docking benchmark version 5 and affinity benchmark version 2. J Mol Biol 2015;427:3031–41. 26231283 10.1016/j.jmb.2015.07.016 PMC4677049

[vbad191-B27] Wang X , Terashi G, Christoffer CW et al Protein docking model evaluation by 3D deep convolutional neural networks. Bioinformatics 2020;36:2113–8. 31746961 10.1093/bioinformatics/btz870 PMC7141855

[vbad191-B28] Wang X , Flannery ST, Kihara D et al Protein docking model evaluation by graph neural networks. Front Mol Biosci 2021;8:647915. 34113650 10.3389/fmolb.2021.647915 PMC8185212

[vbad191-B29] Wei J , Tay Y, Bommasani R et al Emergent abilities of large language models. arXiv, arXiv:2206.07682, 2022, preprint: not peer reviewed.

[vbad191-B30] Xue LC , Dobbs D, Bonvin AMJJ et al Computational prediction of protein interfaces: a review of data driven methods. FEBS Lett 2015;589:3516–26. 26460190 10.1016/j.febslet.2015.10.003 PMC4655202

[vbad191-B31] Zhou H , Skolnick J. GOAP: a generalized orientation-dependent, all-atom statistical potential for protein structure prediction. Biophys J 2011;101:2043–52. 22004759 10.1016/j.bpj.2011.09.012 PMC3192975

[vbad191-B32] Zhu W , Shenoy A, Kundrotas P et al Evaluation of AlphaFold-Multimer prediction on multi-chain protein complexes. Bioinformatics 2023;39:btad424. 10.1093/bioinformatics/btad424 PMC10348836 37405868

