# Supplementary material for: DeepRank-GNN-esm: a graph neural network for scoring protein–protein models using protein language model
Source: Bioinform Adv. 2024 Jan 5;4(1):vbad191. doi: 10.1093/bioadv/vbad191 (PMC10782804; doi:10.1093/bioadv/vbad191)

# Supplementary Material

# DeepRank-GNN-esm: A Graph Neural Network For Scoring Protein-Protein Models With Protein Language Model

X. Xu[1], A. M. J. J. Bonvin[1,*]

[1]Computational Structural Biology Group, Department of Chemistry, Bijvoet Centre, Faculty of Science, Utrecht University

*To whom correspondence should be addressed: a.m.j.j.bonvin@uu.nl

**Supplementary Method 1.** Details of ESM-2 embeddings calculations ........ 2

**Supplementary Method 2.** Definition of the six machine learning metrics used for assessing the performance ........ 3

**Supplementary Table S1.** Data composition of the training, evaluation, and test sets ........ 4

**Supplementary Table S2.** Computing time requirements comparison between PSSM and esm features ........ 4

**Supplementary Figure S1.** Losses and AUC curves for the DeepRank-GNN-esm-pssm models during cross-validation ........ 5

**Supplementary Figure S2.** Losses and AUC curves for the DeepRank-GNN-esm model during cross-validation ........ 6

**Supplementary Figure S3.** Losses and AUC curves for the DeepRank-GNN-no-pssm model during cross-validation ........ 7

**Supplementary Figure S4** Losses and AUC curves for three final models ........ 8

**Supplementary Figure S5.** Scatter plots of Fnat versus predicted Fnat for the four models on BM5 evaluation set ........ 9

**Supplementary Figure S6.** Losses and AUC curves of two models trained on MANY dataset ........ 10

**Supplementary Method 1.** Details of ESM-2 embeddings calculations

We first extracted the sequence for each chain (A, B) in all protein-protein complexes. To compute esm-2 embeddings for those sequences in bulk, we used the python script (`extract.py`) provided at https://github.com/facebookresearch/esm with the command below:

```
python extract.py esm2_t33_650M_UR50D all.fasta ../ -repr_layers 0 32 33 --include mean per_tok
```

In the above command, "`esm2_t33_650M_UR50D`" specifies the pre-trained model to be used for the embeddings. We chose to include embeddings from layers 0, 32, and 33 by using the '`repr-layers`' option. The '`include`' option was used to generate an averaged embedding per amino acid per layer over the entire protein sequence. This means that for each layer, the script computed an embedding for every amino acid in the sequence and then averaged those embeddings to obtain a single representation per amino acid per layer. As a result of this process, the script generated one `.pt` file per FASTA sequence. Each `.pt` file contains the embeddings for each residue in the protein sequence. Subsequently, these generated embeddings were associated with nodes in protein interface graphs and incorporated into the HDF5 files used by DeepRank-GNN.

**Supplementary Method 2.** Definition of the six machine learning metrics used for assessing the performance

1. Precision measures the accuracy of positive predictions made by the model as such:

$$Precision = \frac{TP}{(TP+FP)} \quad (1)$$

2. MCC (Matthews's correlation coefficient) quantifies the overall quality of binary classifications, considering true and false positives and negatives as such:

$$MCC = \frac{(\mathrm{TP} * \mathrm{TN} - \mathrm{FP} * \mathrm{FN})}{\sqrt{(\mathrm{TP} + \mathrm{FP}) * (\mathrm{TP} + \mathrm{FN}) * (\mathrm{TN} + \mathrm{FP}) * (\mathrm{TN} + \mathrm{FN}))}} \quad (2)$$

3. Recall calculates the proportion of actual positives correctly identified by the model as such:

$$Recall = \frac{TP}{(TP+FN)} \quad (3)$$

4. F1 Score combines precision and recall to provide a balanced measure of model performance as such:

$$\mathrm{F1\ Score} = \frac{2 * (\mathrm{Precision} * \mathrm{Recall})}{(\mathrm{Precision} + \mathrm{Recall})} \quad (4)$$

5. $R^2$ score measures the proportion of the variance in the dependent variable that can be explained by the independent variable(s) as such:

$$R^2 = 1 - \frac{\sum_i (y_i - \hat{y}_i)^2}{\sum_i (y_i - \bar{y}_i)^2} \quad (5)$$

6. The Pearson correlation coefficient quantifies the strength and direction of the linear relationship between two variables as such

$$r = \frac{\sum (x_i - \bar{x})(y_i - \bar{y})}{\sqrt{\sum (x_i - \bar{x})^2 \sum (y_i - \bar{y})^2}} \quad (6)$$

**Supplementary Table S1.** Data composition of the training, evaluation, and test sets

| Dataset | Number of complexes | Total | Positive | Negative | Percentage of positive models |
|---|---|---|---|---|---|
| BM5_train | 127 | 2298799 | 166893 | 2131906 | 0.0726 |
| BM5_eval | 127 | 559664 | 40373 | 519291 | 0.0721 |
| BM5_test | 15 | 338995 | 19459 | 319536 | 0.0574 |
| CAPRI_test | 13 | 16593 | 1968 | 14625 | 0.1186 |

**Supplementary Table S2.** Computing time requirements comparison between PSSM and esm features

| Model | Feature Generation Time per model | Average Training Time per epoch (hour) | Average Inference Time per epoch (hour) | Average Inference Time per model (second) |
|---|---|---|---|---|
| DeepRank-GNN-esm-pssm | 2 hours | 30.97 | 2.52 | 0.016 |
| DeepRank-GNN-esm | 5 seconds | 29.30 | 2.45 | 0.015 |
| DeepRank-GNN | 2 hours | 24.80 | 2.35 | 0.015 |
| DeepRank-GNN-no-pssm | 5 seconds | 8.64 | 1.52 | 0.009 |

*All models were trained on a GeForce GTX 1080 Ti.

**Supplementary Figure S1.** Losses and AUC curves for the DeepRank-GNN-esm-pssm models during cross-validation

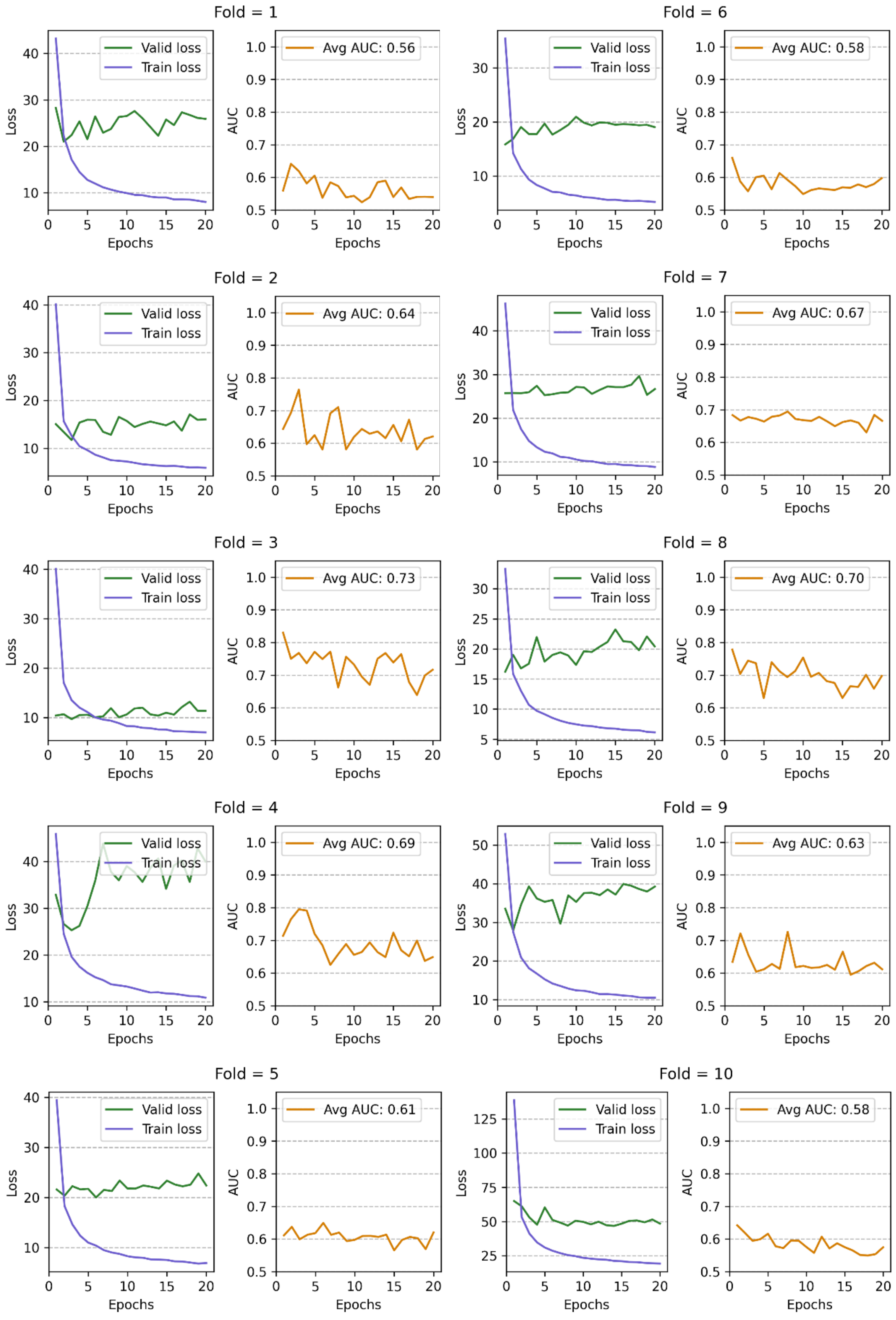

**Supplementary Figure S2.** Losses and AUC curves for the DeepRank-GNN-esm model during cross-validation

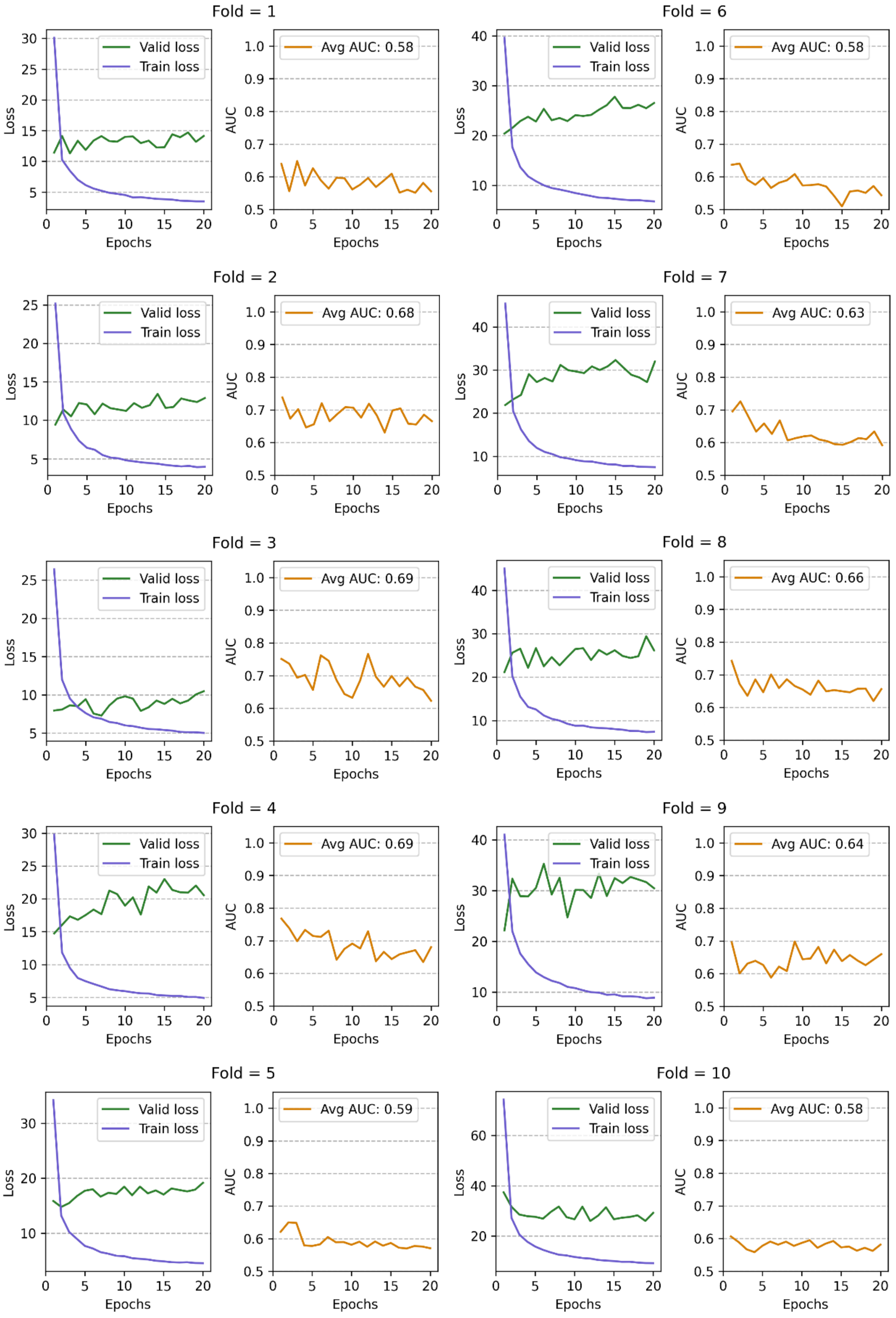

**Supplementary Figure S3.** Losses and AUC curves for the DeepRank-GNN-no-pssm model during cross-validation

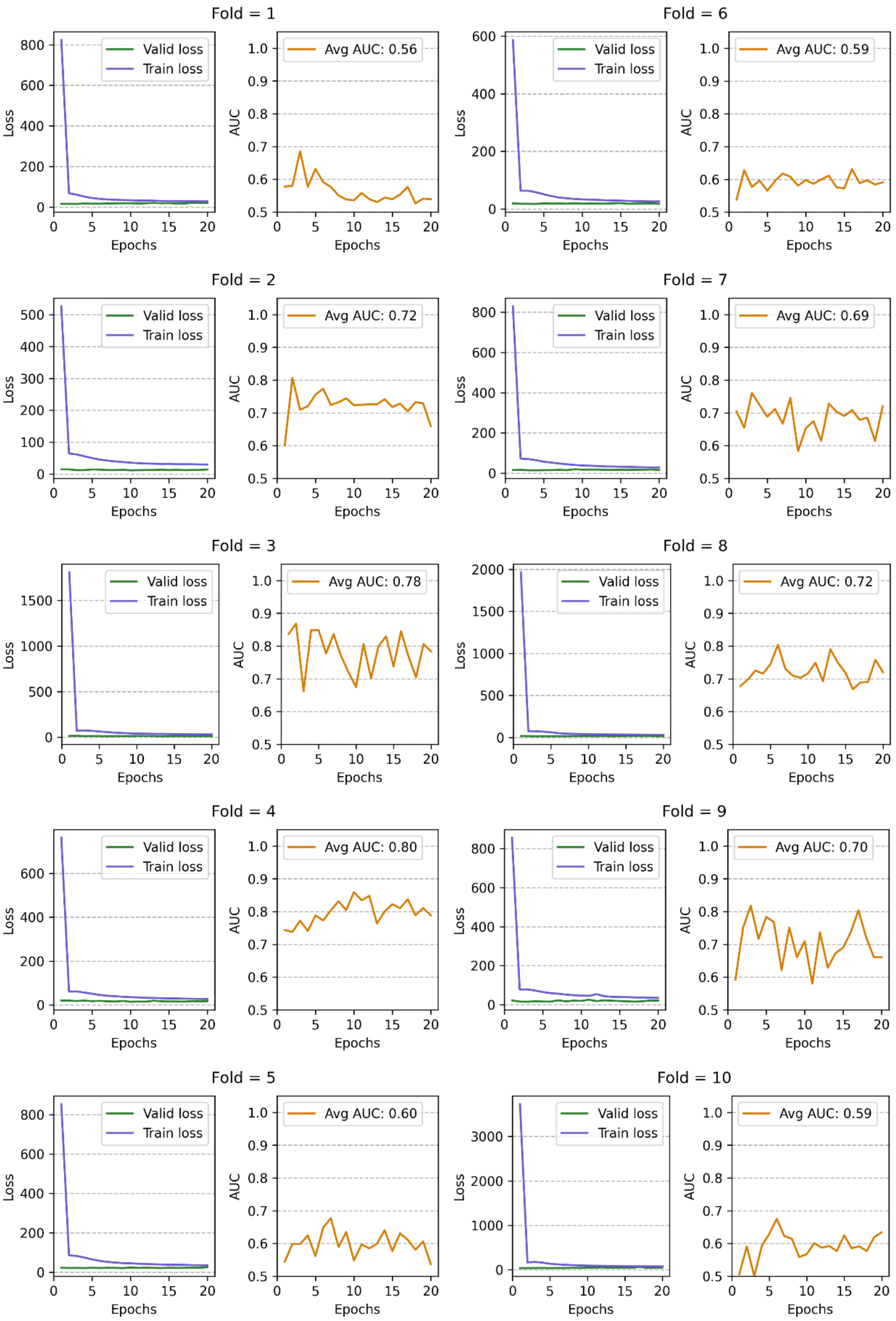

**Supplementary Figure S4** Losses and AUC curves for three final models

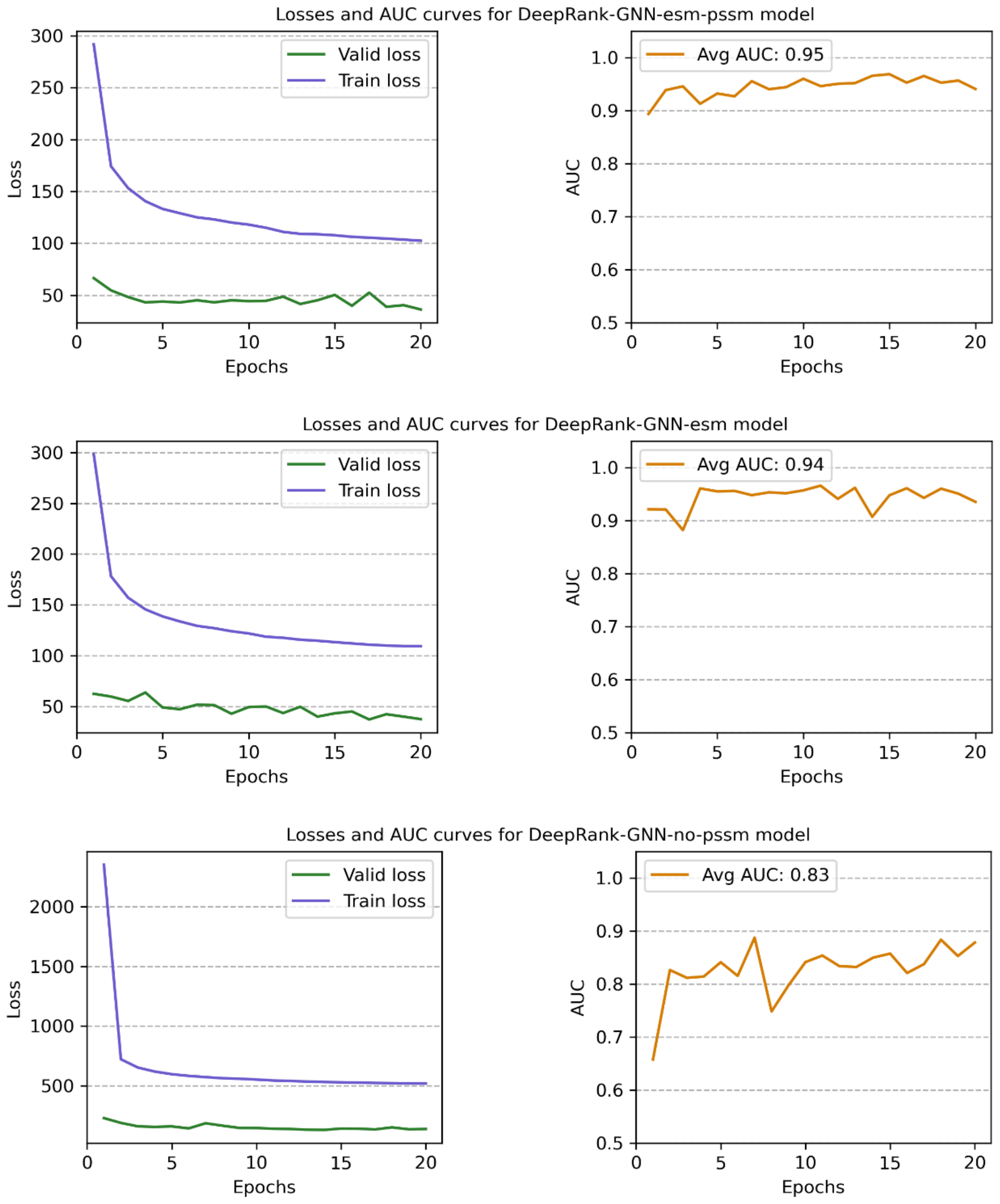

**Supplementary Figure S5.** Scatter plots of Fnat versus predicted Fnat for the four models on BM5 evaluation set

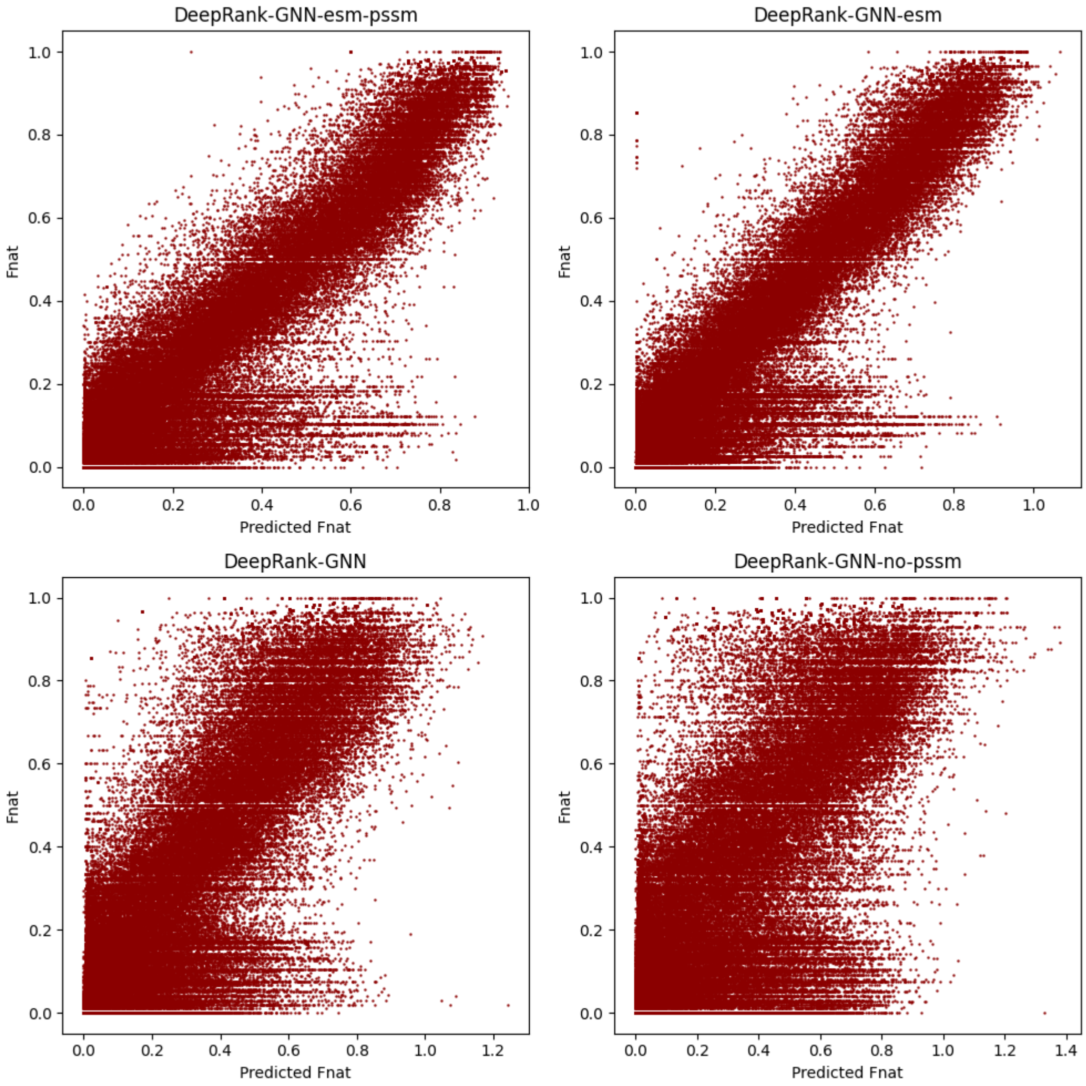

**Supplementary Figure S6.** Losses and AUC curves of two models trained on MANY dataset

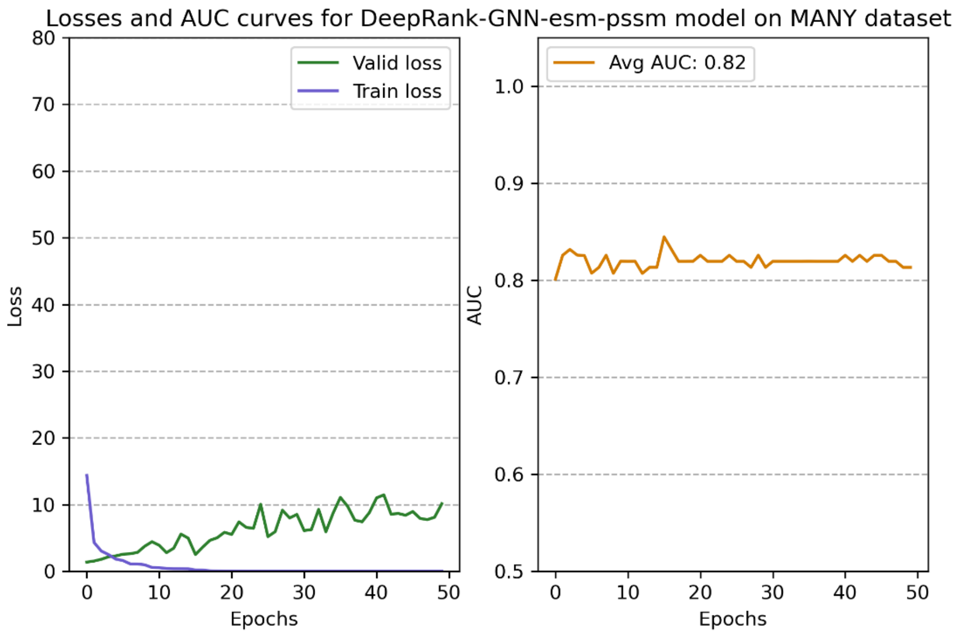


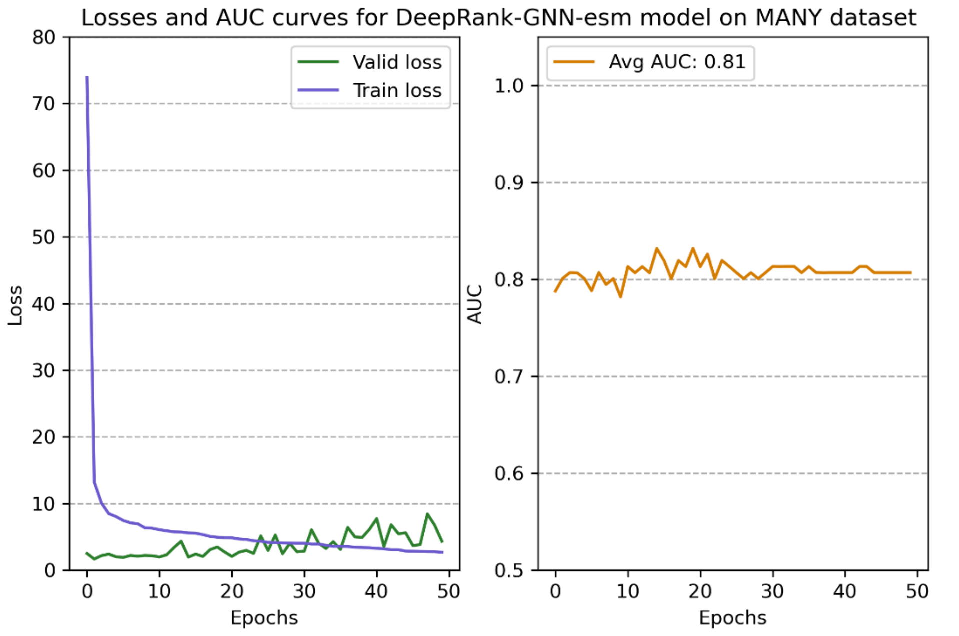

Supplement: vbad191_Supplementary_Data [file vbad191_supplementary_data.pdf]
